# Near-Infrared Fluorescence Imaging for Intraoperative Perfusion Assessment of the Bone During Elective Non-union Surgery: A Feasibility Study

**DOI:** 10.2106/JBJS.OA.25.00177

**Published:** 2026-04-07

**Authors:** S. Koning, D.F. Boldewijn, S.W.R. Dalmeijer, B.E. Zweedijk, M.W. Kruiswijk, R.C. Peul, L.E. van der Aa, I. Planting, F.P. Tange, P. Hoven van den, D.E. Hilling, S. Keereweer, A.L. Vahrmeijer, M.H.J. Verhofstad, J.F. Hamming, I.B. Schipper, M.M.E. Wijffels, J.R. van der Vorst

**Affiliations:** 1 Department of Surgery, Leiden University Medical Center, Leiden, the Netherlands; 2 Department of Otorhinolaryngology and Head and Neck Surgery, Erasmus MC Cancer Institute, Rotterdam, the Netherlands; 3 Department of Surgical Oncology and Gastrointestinal Surgery, Erasmus MC Cancer Institute, Rotterdam, the Netherlands; 4 Trauma Research Unit, Department of Surgery, Erasmus MC, University Medical Center Rotterdam, Rotterdam, the Netherlands

## Abstract

**Background:**

Fracture non-union cause chronic pain, dysfunction, and high healthcare costs. Treatment consists of revitalizing the nonhealed fracture site through debridement, bone resection, grafting, or stabilization if indicated. Adequate bone perfusion is essential for healing, but current intraoperative assessments are subjective. This study explores the feasibility of using quantitative near-infrared fluorescence (NIRF) imaging with indocyanine green (ICG) to assess bone perfusion in non-union surgery. This technique allows real-time visualization of tissue perfusion through tracing intravascular injected ICG.

**Methods:**

This prospective, multicenter feasibility study was conducted in 2 Dutch academic hospitals. Intraoperative ICG NIRF imaging was performed in non-union patients after debridement and before osteosynthesis. Time-intensity curves were derived from intraoperative data, and 3 time-related perfusion parameters were extracted to quantify the perfusion in various osseous structures. Demographic data included patient's age, sex, race, and body mass index.

**Results:**

ICG NIRF measurement was successfully performed in 20 patients (median age 50.5 years, 65% female, and 90% White) at 7 fracture locations. Three distinct perfusion patterns were observed. (1) rapid ingress and egress, (2) rapid ingress with a plateau phase, and (3) prolonged ingress with no egress within the measurement. The median Tmax was 214 s (inter-quartile range [IQR]): 69-246 s. The normalized maximum ingress slope was 4.4%/s (IQR: 2.4-6.5), and the median Egress-60 was 84.7% (IQR: 77.4-94.8).

**Conclusion:**

ICG NIRF imaging is a feasible and reproducible method for intraoperative bone perfusion assessment in fracture non-union, potentially offering an objective measure of bone vitality. Perfusion parameters and time-intensity curves were successfully quantified across various bone structures, revealing patterns similar to those linked with clinical outcomes in other studies. Future research should evaluate how these perfusion patterns relate to bone healing.

**Level of Evidence:**

Level II. See Instructions for Authors for a complete description of levels of evidence.

## Introduction

Adequate bone perfusion is essential for fracture healing and preventing non-union complications^1,2^. In fracture non-union surgery, intraoperative bone vitality assessment is often subjective, risking inadequate treatment and suboptimal outcomes^3^. Non-unions occur in 5%–10% of all long bone fractures, posing a major challenge in trauma surgery^4,5^.

Non-union patients often experience persistent pain and reduced function^6^. Treatment depends on the non-union type: hypertrophic requires stability, atrophic needs debridement and biological support, aseptic calls for bone grafting, reduction and stabilization, and septic necessitates aggressive management, including infected tissue debridement^7-11^. In all types, removal of interposed fibrous or devitalized tissue is essential to promote successful healing.

Suboptimal debridement or excessive bone resection can worsen non-union, causing chronic pain, functional impairment, and increased healthcare costs due to prolonged treatment and repeated surgeries^3,12^. Overresection of viable bone may compromise structural integrity and limit reconstructive options^3,12-15^.

Currently, perfusion assessment primarily relies on visual and tactile cues, such as color, consistency, capillary bleeding, and the “paprika sign” after cortical drilling^16,17^. Despite their widespread use, these subjective methods lack accuracy and reproducibility, depending on the surgeon's visual estimations, leading to variations in debridement thoroughness^18,19^.

To date, no gold standard exists to evaluate bone perfusion. Techniques, such as PET-computed tomography, dynamic contrast-enhanced magnetic resonance imaging (DCE-MRI) and contrast-enhanced ultrasound, have been explored but are often impractical intraoperatively due to cost, complexity, and limited applicability^20-24^. Therefore, a reliable, objective, and real-time intraoperative perfusion assessment tool is needed to optimize surgical decision-making in non-union fractures.

Near-infrared fluorescence (NIRF) imaging with indocyanine green (ICG) has emerged as a promising technique for real-time perfusion assessment in various surgical fields. ICG, visualized with a NIR camera, enables dynamic visualization of blood flow due to its binding to plasmatic proteins and confinement to the intravascular compartment. Previous studies have shown its effectiveness in assessing soft tissue perfusion, guiding debridement, and predicting wound healing^25-33^. In addition, ICG NIRF imaging has shown potential for intraoperative assessment of bone perfusion^28,34,35^. However, its application in fracture non-union surgery remains largely unexplored.

Therefore, the aim of this study was to evaluate the feasibility of quantitative ICG NIRF imaging for intraoperative bone perfusion assessment in elective non-union surgery, with a focus on its clinical usability.

## Material and Methods

### Participants

This prospective interventional feasibility study (NCT06034834) included adults treated for a non-union and/or a fracture-related infection (FRI) in the Leiden University Medical Center and the Erasmus Medical Center between February 2024 and January 2025. Non-union was defined as a nonhealing fracture existing for at least 9 months^36^. The criteria for a FRI were defined by Metsemakers et al. using extensive clinical, microbiological, radiological, and histopathological criteria as elaborated in their international expert group consensus^37^. Exclusion criteria were derived from the instructions for use from Diagnostic Green, the ICG manufacturer: hyperthyroidism, renal failure with eGFR <30 L/min/1.73 m^2^, severe liver failure, patients who are allergic to ICG, iodine, or shellfish.

Preoperative recorded data included patient's age, sex, race, height, weight, body mass index, medical history, history of smoking, and injury location. Intraoperative data included estimated blood loss and vital signs, recorded at a single time point when the ICG was injected. All data were collected in Castor (CDMS version 2024.4.4.0).

### Surgery and Fluorescence Imaging

**Fig. 1:**
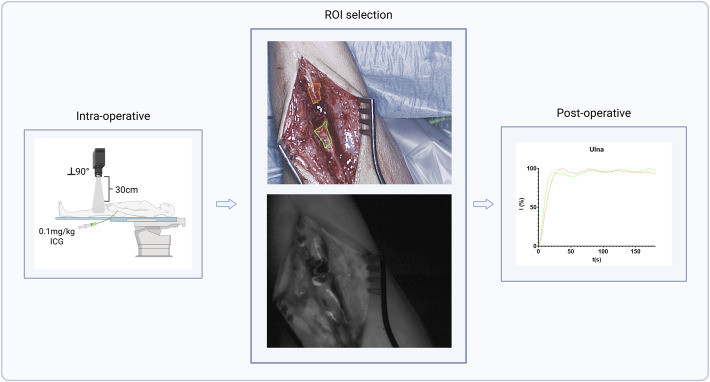
NIRF imaging method separated in the intraoperative, ROI selection, and postoperative phase. Left image depicts the standardized imaging method. Middle upper image displays the ROI selection in an ulna non-union. Middle bottom image displays the NIRF signal after ICG injection. The right image shows the time-intensity curves derived from these ROIs. ICG = indocyanine green, NIRF = near-infrared fluorescence, and ROI = regions of interest.

All eligible patients underwent perfusion assessment using NIRF imaging with ICG, performed by trained personnel after completion of debridement and immediately before reconstruction (osteosynthesis or grafting), as determined by the senior surgeon. A standardized imaging protocol was followed to minimize potential confounding. A 5-minute NIRF-recording was conducted using the Quest Spectrum 2.0 Platform (Quest Medical Imaging) at fixed settings, at 30 cm perpendicular to the plane of the fracture using a measuring tape. Ambient light was turned off and windows blinded to optimize imaging. ICG (Verdye 25 mg) was administered intravenously at 0.1 mg/kg, followed by a 10cc saline flush. The camera system featured a dual light source providing LED white light and a NIR-source emitting at a wavelength of 700 to 820 nm. Fig. 1 shows the complete imaging method used. Surgeons were blinded to the NIRF data.

### Outcomes

The primary outcome was the feasibility of using NIRF imaging for osseous perfusion assessment, defined by the ability to reproducibly extract time-intensity curves and correlated perfusion parameters from the ICG NIRF signal.

### Quantification of the Fluorescent Signal

Although the analyses software is available intraoperatively, the fluorescence videos were deliberately analyzed postoperatively using the Quest Research Framework (Quest Medical Imaging) software. This produces time-intensity curves correlated to the NIRF-signal in the selected regions of interest (ROI), providing extractable inflow and outflow parameters. The ROIs were drawn on both sides of the fracture, as depicted in Fig. 1. The extracted perfusion parameters included:

- Tmax (s): The time until maximum fluorescence intensity, representing the arterial inflow rate. A lower value indicates that the maximum is achieved more quickly.
- Normalized Maximum Ingress Slope (%/s): The steepest segment of the curve on contrast arrival, representing the capacity for arterial perfusion. A higher value indicates more rapid arterial inflow.
- Egress-60(%): The percentage of maximum intensity 60 seconds after Tmax; this is an indicator of tissue retention and venous outflow. A lower value indicates faster venous outflow.

These parameters were calculated using distance-corrected standardized algorithms and curve normalization. The normalization process adjusts the absolute fluorescence signal to account for variables such as camera distance, angle, and intensity fluctuations. This technique scales the fluorescence intensity to a standardized range of 100%, allowing for a more consistent comparison of relative perfusion levels regardless of slight changes in imaging conditions. This method has been validated in prior research^26,32,38-40^. The time-intensity curve with extracted parameters are displayed in Appendix Fig. 1.

### Statistical Analyses

Descriptive statistical analyses were calculated using IBM SPSS 26.0 (IBM corp). Normality was assessed using the Shapiro-Wilk test. Continuous variables were reported as means (SD) or medians (IQR) and categorical variables as frequencies (%). Time-intensity curve generation was conducted using GraphPad Prism (version 10.2.3, GraphPad Software).

### Ethical Considerations

This study (NCT06034834) was approved by the Medical Ethics Committee Leiden The Hague Delft (METC-LDD) and conducted in accordance with the Declaration of Helsinki and institutional guidelines. All patients enrolled provided written informed consent.

## Results

### Patient Characteristics

This study initially comprised 27 patients, of which 7 patients were excluded due to invalid measurements. Six additionally approached patients declined to join the study. Invalid measurements included excessive bleeding at the fracture site (n = 2), obstructing instruments (n = 2), and moving object interference (n = 3) (Appendix Fig. 2). Of the final study cohort (n = 20), 65% were women (n = 13), 90% were White (n = 18), and the overall mean age was 50.5 ± 17.4 years. All patients were diagnosed with and operatively treated for a non-union, 3 (15%) patients were diagnosed with a simultaneous FRI. The median time between fracture and non-union surgery was 16 months (IQR: 12-31.5). Seven different fracture locations were included in this analyses. A complete overview of patient characteristics and procedural details is presented in Table I. Patient characteristics and procedural details of the excluded patients are presented in Appendix Table 1 and Appendix Table 2.

**TABLE I:** Patient and Procedural Characteristics

Demographics	Total (n = 20)
Female*	13 (65%)
Age†	50.5 (17.4)
BMI†	25.6 (4.3)
Hypertension*	3 (15%)
Smoking*—Active	2 (10%)
History of smoking*	5 (25%)
Diabetes*	0
FNO time‡	16 [12–31.5]
Procedural details	
Trauma*	
Non-union	20 (100%)
Fracture-related infection	3 (15%)
Location of injury* (%)	
Humerus	3 (15)
Clavicula	5 (25)
Scaphoid	1 (5)
Costa	2 (10)
Femur	5 (25)
Tibia	3 (15)
Ulna	1 (5)
Intraoperative measurements	
Pulse OR†	67.9 (13.7)
Systolic tension*	110.2 (18.6)
Diastolic tension†	61 (10.1)
Blood loss‡	200 [0-600]
Length of hospital stay*	1 [0-3]

BMI = body mass index, FNO-time = time between fracture and non-union operation in months, and OR = operating room.

* Variables are denoted as n (%).

† Variables are denoted as mean (SD).

‡ Variables are denoted as median [interquartile range].

### Time-intensity Curves

**Fig. 2:**
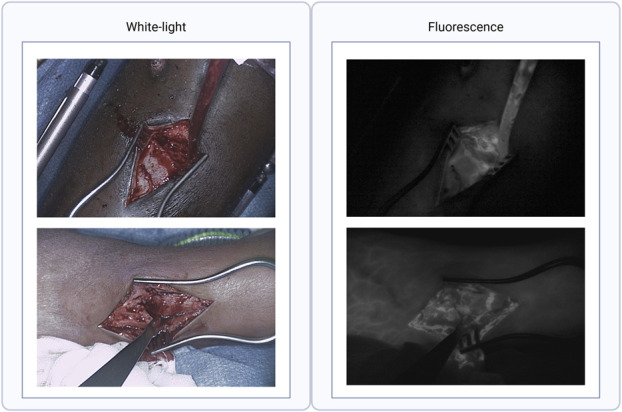
Two examples of the fluorescent ICG signal in 2 different anatomical locations. The left panels display white light images of the fracture. The right panel show the fluorescent ICG signal captured by the NIR camera. ICG = indocyanine green, and NIR = near-infrared.

**Fig. 3:**
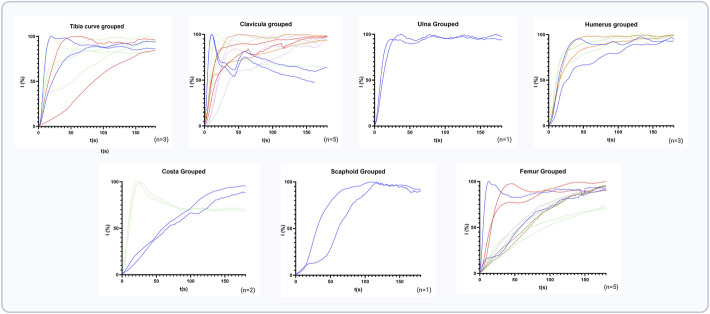
All time-intensity curves categorized per bone type. Each color within a graph represents 1 patient. Each line represents a separate ROI drawn within the fracture. Signal intensity in percentages is presented on the y-axis, and time in seconds is presented on the x-axis. ROI = regions of interest.

**Fig. 4:**
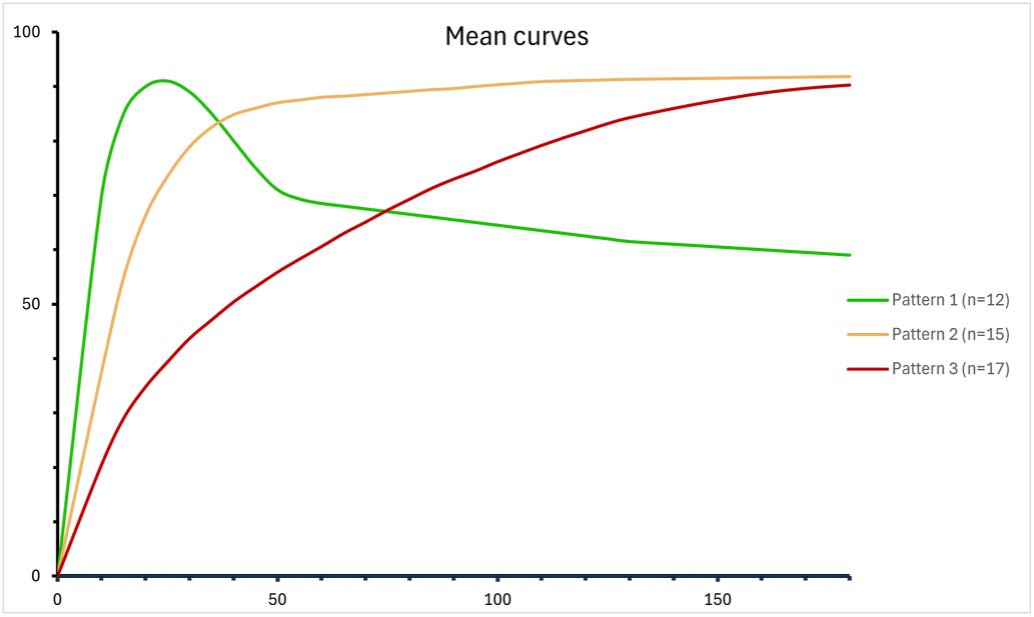
Mean observed patterns across all ROIs categorized in 3 patterns. In green pattern 1, in yellow pattern 2, and in red pattern 3. Signal intensity in percentages is presented on the y-axis, and time in seconds is presented on the x-axis. ROI = regions of interest.

Fig. 2 shows the NIRF-signal captured in 2 different anatomical locations. Fig. 3 presents all time-intensity curves specified per bone. Three distinct perfusion patterns were observed, characterized by different inflow and outflow patterns (Fig. 4). These patterns were derived from those previously described by Tange et al^41^.

Pattern 1 was characterized by a steep ingress slope, followed by a steep egress slope. Pattern 2 shows a steep ingress slope that reaches a plateau phase. Pattern 3 shows a prolonged ingress during which the egress has not been reached.

### Quantitative Analysis

Appendix Table 3 presents the quantified perfusion parameters for all bones collectively categorized per bone location. The median Tmax for all bones collectively was 214 s (IQR: 69-246 s), with the shortest median Tmax observed in the scaphoid at 98.5 s (IQR: 93-NA), and the longest median Tmax recorded in the femur at 231 s (200.5-274.3). The normalized maximum ingress slope was 4.4%/s (IQR: 2.4-6.5) with the steepest slope observed in the ulna at 6.9%/s (IQR: 6-NA) and the flattest slope in the femur at 1.7%/s (1.4-4.4). The egress phase showed a median Egress-60 of 84.7% (IQR: 77.4-94.8), with the lowest Egress-60 in the humerus at 99% (98.9-NA) and highest in the costa at 82.6% (82.3-NA).

## Discussion

This study demonstrates that ICG NIRF imaging is a feasible and reproducible technique for intraoperative perfusion assessment in fracture non-unions. By extracting time-intensity curves, including inflow and outflow parameters, this method enables quantitative analysis of bone perfusion. Importantly, its feasibility across different bone structures and patient cases highlights its potential for broader clinical applicability.

Prior research has validated ICG NIRF imaging for soft tissue perfusion across various surgical fields^25-27,30,32,33,41^. In orthopaedic trauma surgery, ICG has been used to assess perfusion in high-risk fractures and amputations, predicting wound healing^28,42^. However, its application in bone perfusion is limited. Various studies demonstrated the utility of NIRF imaging in assessing bone viability using dynamic perfusion parameters^18,21,23,24,35^. Valerio et al. used maximum signal intensity compared with a reference region, but this method is influenced by variables such as camera distance, angle, and injection dosage, lacking inflow or outflow parameters^24,32^. Gitajn et al. and Elliott et al. able to extract time-intensity curves from bone using a kinetic flow model quantifying the signal^18,21^. However, both studies were performed in a small number of porcine subjects which are known to have different osseous properties^43^. Our study builds on these findings by implementing a standardized and validated quantification technique in a prospective human cohort. By correcting for variables such as signal intensity, camera distance, and tissue angle, through normalization, thereby improving clinical applicability.

Interestingly, our study identified 3 distinct intraoperative fluorescence perfusion patterns, closely resembling the findings reported by Galema et al. in gastric conduit assessment^33^. They stratified perfusion patterns into 3 zones: Pattern 1 represented well-perfused tissue at the base of the conduit, clinically regarded as viable; Pattern 2 indicated a transition zone; and Pattern 3 corresponded to the ischemic tip of the conduit^33^. Our findings show similar fluorescence profiles, although due to the small cohort size, no conclusions on clinical implications or outcomes can be drawn.

In addition, variations in perfusion characteristics were observed across different anatomical locations, underscoring the need to investigate the relationship between bone type, vascularity, and fracture healing. These differences likely stem from anatomical and physiological variability in skeletal vascularization, as well as trauma-induced disruption of local blood supply^44,45^. For instance, the tibial diaphysis is known for its relatively sparse vascular network compared with the more richly perfused clavicle. This comparison highlights the biological differences in vascularity, rendering the former more susceptible to non-union development^46,47^. These anatomical differences may explain the observed variations in fluorescence intensity and perfusion kinetics.

The strengths of this study include a standardized imaging protocol, quantitative analysis, and evaluation of multiple bone structures, providing a comprehensive analysis of how ICG NIRF imaging performs in different anatomical contexts. In addition, the standardized approach minimized variability, ensuring consistent imaging conditions across patients, while the quantitative analysis provided an objective measure of perfusion. The multicenter design enhances the generalizability of our findings, suggesting that ICG NIRF imaging can be reliably applied in diverse clinical settings.

In the initial phase, 7 patients were excluded due to technical or methodological limitations, highlighting practical challenges and the need for refinement. The most common issues included excessive bleeding, suboptimal bone exposure, and interference from fixation materials, which hindered reliable fluorescence assessment. These early challenges prompted led to a more rigorous standardization of the imaging procedure in subsequent cases to improve signal consistency and interpretability. Future studies should build on these experiences by further controlling for these variables, to refine the methodology and improve the applicability. Finally, real-time intraoperative analysis is technically feasible, but this study focused on postoperative analysis as a feasibility assessment without influencing clinical decisions. Future research will explore integrating real-time analysis into the surgical workflow, enabling informed decisions on tissue removal and reconstructive interventions.

Our findings demonstrate that ICG NIRF imaging is a feasible and reproducible tool for assessing bone blood flow in fracture non-unions. This real-time intraoperative technique aids tissue viability evaluation. If future studies link perfusion parameters with clinical outcomes, it could improve surgical decision-making, reduce unnecessary bone resection, ensure nonviable tissue removal, and potentially lower complications and reinterventions.

## Conclusion

This study demonstrates that ICG NIRF imaging is a feasible, reproducible method for intraoperative bone perfusion assessment in fracture non-unions. We successfully quantified time-intensity curves and perfusion parameters, confirming its applicability across different bone structures. These findings lay the groundwork for exploring the link between perfusion parameters and long-term bone healing, with the goal of integrating ICG NIRF imaging into routine non-union fracture treatment. This technique offers real-time, objective bone viability assessment, which could improve surgical decisions, optimize outcomes, and reduce healthcare costs.

## Funding

This project is cofunded by the PPP Allowance made available by Health∼Holland regarding the RULER and IMPULSE-project. Top Sector Life Sciences & Health, to stimulate public-private partnerships. Together with the RETINA-project receiving funding from the European Union's Horizon Europe research and innovation program under grant agreement number 101135529.

## Appendix

Supporting material provided by the authors is posted with the online version of this article as a data supplement at jbjs.org (http://links.lww.com/JBJSOA/B139). This content was not copyedited or verified by JBJS.
